# MentalRoBERTa-Caps: A capsule-enhanced transformer model for mental health classification^☆^

**DOI:** 10.1016/j.mex.2025.103483

**Published:** 2025-07-09

**Authors:** Faheem Ahmad Wagay, Jahiruddin, Yasir Altaf

**Affiliations:** a Jamia Millia Islamia University, New Delhi 10025, India; b Maulana Azad National Urdu University, Gachibowli, Hyderabad 500032, India

**Keywords:** Bert, Capsule, Reddit, Roberta, Mental illness

## Abstract

In recent years, the dominance of Large Language Models (LLMs) such as BERT and RoBERTa has led to remarkable improvements in NLP tasks, including mental illness detection from social media text. However, these models are often computationally intensive, requiring significant processing time and resources, which limits their applicability in real-time or resource-constrained environments. This paper proposes a lightweight yet effective hybrid model that integrates a 6-layer RoBERTa encoder with a capsule network architecture to balance performance, interpretability, and computational efficiency. The contextual embeddings generated by RoBERTa are transformed into primary capsules, and dynamic routing is employed to generate class capsule outputs that capture high-level abstractions.

To validate performance and explainability, we employ LIME (Local Interpretable Model-Agnostic Explanations) to provide insights into feature contributions and model decisions. Experimental results on benchmark mental health datasets demonstrate that our approach achieves high accuracy while significantly reducing inference time, making it suitable for deployment in real-world mental health monitoring systems.

1. To design a computationally efficient architecture for mental illness detection using a lightweight RoBERTa encoder integrated with capsule networks.
2. To perform a detailed time complexity analysis highlighting the trade-offs between performance and efficiency.
3. To enhance model interpretability through LIME-based feature attribution, supporting transparent and ex- plainable predictions.

## Graphical abstract

**Figure:**
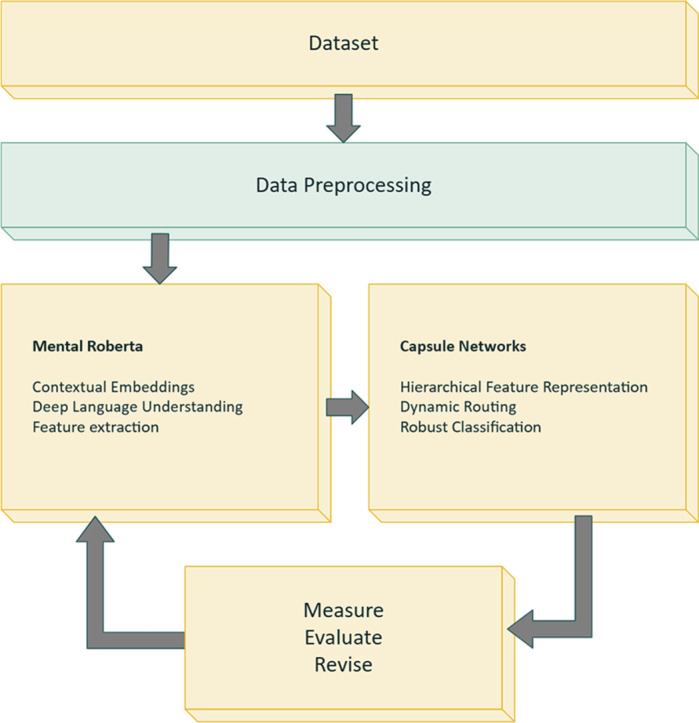

## Specifications table

**Table:** 

**Subject area**	*Computer Science*
**More specific subject area**	*Artificial Intelligence*
**Name of your method**	*MentalRoBERTa-Caps: A Capsule-Enhanced Transformer Model for Mental Health Classification*
**Name and reference of original method**	*Z. Ji, Z. Zhou, T. He, G. Bajaj, J. Ye, Y. Sun, W. Xu, P. Zhao, Q. Le, Q. Tran,* et al.*, “Mentalbert: Publicly available pretrained language models for mental healthcare,” in Proceedings of the 60th Annual Meeting of the Association for Computational Linguistics: System Demonstrations, pp. 107–118, 2021.*
**Resource availability**	*Code link will be provided after acceptance.*

## Background

Mental illness detection from social media text has emerged as a critical area of research at the intersection of natural language processing (NLP) and healthcare. Early approaches relied heavily on traditional machine learning models such as Support Vector Machines (SVMs) and Na¨ıve Bayes classifiers, using handcrafted features to classify mental health conditions. However, with the rise of deep learning, particularly transformer-based models, the field has witnessed substantial improvements in classification accuracy and feature representation. The introduction of BERT (Bidirectional Encoder Representations from Transformers) [1] brought a paradigm shift in NLP by enabling deep bidirectional representations. Several studies have adopted BERT and its domain-specific variants to detect mental health conditions such as depression, anxiety, and stress. For example[2], utilized BERT to extract contextual features from Reddit posts for binary depression classification, showing notable improvements over traditional feature- engineering-based methods. Similarly Murarka et al. [4] uses Roberta for mental health detection. To better capture the unique linguistic and psychological patterns inherent in mental health discourse, researchers have developed domain-specific models. Notably, MentalBERT and MentalRoBERTa [3] were pretrained on large-scale mental health-related corpora sourced from Reddit, enabling more effective recognition of emotional and cognitive cues in social media text. Expanding on these efforts, the development of MentaLLaMA [5] represents a recent milestone in the field. Trained on instruction-tuned mental health datasets, MentaLLaMA was designed to provide both predictions and interpretable explanations. However, despite its interpretability and task coverage, its classification metrics still lag slightly behind the performance of **MentalRoBERTa** [4], Despite their high accuracy, BERT and its extended models are computationally expensive, often requiring significant memory and processing time. This hinders their applicability in low-resource settings or in scenarios demanding real-time analysis. Capsule Networks, introduced by Sabour et al. [6], offer a novel way to model hierarchical relationships in data using vector-based capsules instead of scalar neurons. In text classification, Capsule Networks have been applied to capture compositional semantics more effectively than CNNs or RNNs. Works such as [7,8] demonstrate the efficacy of Capsule Networks in sentiment analysis and intent detection tasks, where spatial hierarchies in text are critical. While Capsule Networks have shown promise in various classification tasks, their integration with transformer-based models remains under-explored, especially in the context of mental illness detection. Combining the contextual strength of transformers like RoBERTa with the representational power of capsules could offer both accuracy and interpretability benefits. Given the growing concern over the time and resource demands of LLMs, our work proposes a hybrid architecture that combines a truncated 6-layer RoBERTa encoder with a Capsule Network for classification. This design aims to retain the contextual understanding of transformer models while leveraging the efficient, hierarchical modeling capacity of capsules, making it a more practical solution for mental health analysis at scale.

## Method details

This section describes the architecture used for mental illness detection, which combines a lightweight RoBERTa en- coder with a capsule-based classification layer as shown in Fig 2. The model processes text in several stages: preprocessing, tokenization and embedding, transformer encoding, capsule transformation, and classification. Each stage is described below in detail.

### Data preprocessing

**Fig. 1:**
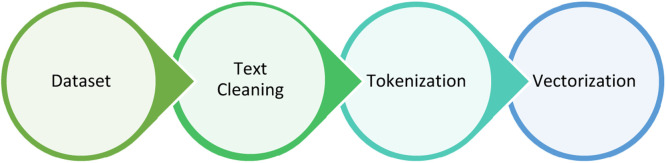
Layers of data preprocessing.

The noisy nature of social media or online mental health data, robust preprocessing is critical. Our preprocessing pipeline shown in Fig 1 is tailored to the requirements of transformer- based models, particularly MentalRoBERTa. The steps are as follows:

• **Text Cleaning:** Remove URLs, user mentions (e.g., @user), special characters, HTML tags, and extra whites- pace. This reduces noise and prevents non-informative tokens from degrading model performance.
• **Tokenization:** We use the WordPiece tokenizer from the HuggingFace Transformers library. This tokenizer breaks words into subword units, allowing efficient handling of rare or domain-specific terms.
• **Transformer Inputs:** The tokenized sequence is converted into:

**Input IDs:** Integer indices corresponding to subword tokens.
**Attention Masks:** Binary masks indicating valid tokens vs. padding.
**Segment IDs (if needed):** Not used in RoBERTa but included for compatibility.

**Fig. 2:**
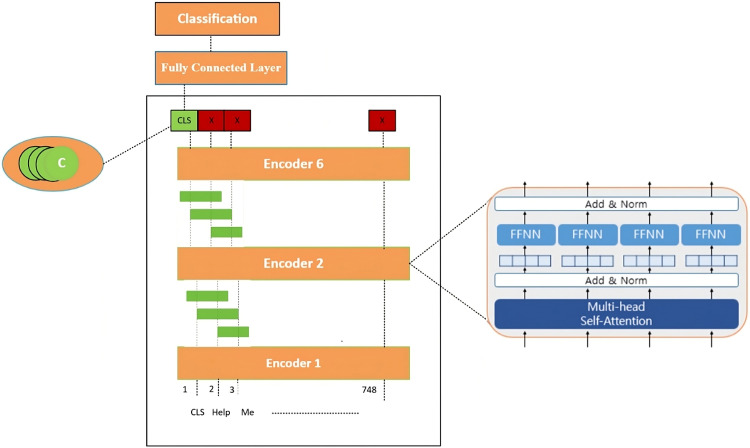
General architecture of our proposed model.

### Input representation

The input sequence be denoted as:

(1) $$x=\left[x_{1},x_{2},\ldots ,x_{n}\right]$$

where x_i_ is the i th token in the input sentence and n is the maximum sequence length.

(2) $$H^{\left(0\right)}=\left[e_{1},\ldots .e_{n}\right]\in R^{n\times d}$$

Each token in the sequence is mapped into a high-dimensional continuous vector e_i_ through contextual embedding, utilizing the RoBERTa tokenizer and embedding layers. These embeddings integrate both semantic content (word identity) and positional information (word order) as follows:

(3) $$e_{i}=E_{w}\left[x_{i}\right]+E_{p}\left[i\right],e_{i}\in R^{d}$$

Here, **E_w_** is the word embedding matrix, **E_p_** is the positional embedding, and d is the hidden size.

The embedding matrix H⁽⁰⁾ of vectors [e_1_….e_n_] is then fed into a stack of Transformer encoder layers, specifically using 6 layers of the RoBERTa architecture. Each Transformer layer consists of two main components:

***Multi-Head Self-Attention*:** Self-attention allows each token to attend to every other token in the sequence, thereby capturing contextual relationships between words, regardless of their positions.

For each head h, the attention is computed as:

(4) $$Q_{h}=H^{\left(l-1\right)}W_{h}^{Q},K_{h}=H^{\left(l-1\right)}W_{h}^{K},V_{h}=H^{\left(l-1\right)}W_{h}^{V}$$

(5) $$O_{h}=Attention\left(Q_{h},K_{h},V_{h}\right)=soft\max\left(\left(Q_{h}K_{h}^{T}\right)/sqrt\left(d_{k}\right)\right)V_{h}$$

Where:

• $W_{h}^{Q}$, $W_{h}^{k}$, $W_{h}^{v}$are learnable weight matrices.
• dₖ is the dimensionality of each head (usually d / number of heads).
• The softmax scores determine how much attention each token should pay to others.

The output of all heads is concatenated and projected:

(6) $$MHSA\left(H^{\left(l-1\right)}\right)=Concat\left(O_{1},\ldots ,O_{H}\right)W^{O}$$

#### Add & norm: first residual block

A residual connection is added to the attention output followed by layer normalization:

(7) $$Z^{\left(l\right)}=LayerNorm\left(H^{\left(l-1\right)}+MHSA\left(H^{\left(l-1\right)}\right)\right)$$

*Feedforward Network (FFN****):*** Each token’s vector is passed independently through a two-layer feedforward network with a non-linear GELU activation:

(8) $$FFN\left(z\right)=GELU\left(zW_{1}+b_{1}\right)W_{2}+b_{2}$$

#### Second residual connection and normalization

(9) $$H^{\left(l\right)}=LayerNorm\left(Z^{\left(l\right)}+FFN\left(Z^{\left(l\right)}\right)\right)$$

The computation is repeated for *L* = 1, …, 6 layers. The final output is:

(10) $$H^{\left(6\right)}=\left[h_{\left[CLS\right]}^{\left(6\right)},h_{2}^{\left(6\right)},\ldots ,h_{n}^{\left(6\right)}\right]\in R^{n\times d}$$

#### Capsule transformation layer

To enhance hierarchical feature learning, we project the output of the transformer [CLS] into multiple primary capsules. Each capsule is expected to capture distinct patterns such as linguistic cues of specific mental illnesses.

#### Primary capsule generation

Each token’s representation **hᵢ ∈ ℝᵈ** from the Transformer is linearly projected into **P** primary capsules using distinct weight matrices:

$$u_{p}=Squash\left(W_{p}h_{i}+b_{p}\right),\forall i\in \left[1,n\right],p=1,\ldots ,P$$

(11) $$W_{p}\in R^{d_{p}\times d},u_{p}\in R^{d_{p}}$$

• W_p_ ∈ ℝᵈᵖˣᵈ is the projection matrix for capsule p.
• b_p_ ∈ ℝᵈᵖ is the corresponding bias.
• u_p_ ∈ ℝᵈᵖ is the output vector of the primary capsule.
• The **Squash** function is a non-linear normalization that ensures shorter vectors get shrunk and longer vectors get slightly shrunk to keep outputs between 0 and 1:
  (12) $$Squash\left(s\right)=\left({\left|\left|s\right|\right|}^{2}/\left(1+{\left|\left|s\right|\right|}^{2}\right)\right)\cdot \left(s/\left|\left|s\right|\right|\right)$$

This operation encourages the magnitude of the capsule output to represent the probability of feature presence, while the orientation encodes its properties.

#### Vote generation and dynamic routing

Each primary capsule **uᵢ** votes for a set of class capsules (representing different mental health conditions). A vote vector is computed:

(13) $$\hat{u_{j|i=W_{ij}\cdot u_{i}}}$$

Where: Wᵢⱼ transforms the output of capsule **i** to predict what it believes capsule j (class) should be.

The dynamic routing process aggregates and iteratively refines the weights (routing coefficients cᵢⱼ) between primary and class capsules. At each iteration:

(14) $$s_{j}=\sum_{i_{ij}^{c}}\hat{u_{j|i}}$$

(15) $$v_{j}=\text{Squash}\left(s_{j}\right)$$

Output**:** The length of each class capsule V_J_ represents the probability of that class:

#### Final classification layer

The outputs from the **C** class capsules are collected:

(16) $$\left[v^{1},v^{2},\ldots ,v_{C}\right]$$

These are concatenated or flattened and passed through a fully connected (dense) layer to make the final prediction.

(17) $$Output=soft\max\left(W_{c}ls\cdot \left[v^{1};v^{2};\ldots ;v_{C}\right]+b_{c}ls\right)$$

Where:

• $W_{c}ls$and $b_{c}ls$ are learnable parameters.
• The **softmax** activation converts raw scores into a probability distribution over **C** classes.
• The predicted class is selected as:
  (18) $$\hat{y}=\arg\max_{j}∥v_{j}∥$$

This classification head leverages the structured, capsule-based representation to make robust and interpretable predictions. This setup enables the model to assign a likelihood to each class based on the hierarchical and spatial features learned by the capsule network, thereby enhancing classification performance in complex, multi-class settings.

### Computational complexity

Let:

n: input sequence length, d: hidden size of Transformer, d_p_: primary capsule dimension, d_c_: class capsule dimension, P: number of primary capsules, C: number of class capsules, R: number of routing iterations

We analyze the overall time complexity to assess the computational feasibility of the model:

RoBERTa Encoder
  (19) $$O\left(6\cdot \left(n^{2}\cdot d+n\cdot d^{2}\right)\right)$$
Primary Capsule Transformation:
  (20) $$O\left(n\cdot d\cdot d_{p}\right)$$
Class Capsules and Routing:
  (21) $$O\left(P\cdot C\cdot d_{p}\cdot d_{c}+R\cdot P\cdot C\cdot d_{c}\right)$$
Total Complexity:
  (22) $$O\left(6\cdot \left(n^{2}\cdot d+n\cdot d^{2}\right)+n\cdot d\cdot d_{p}+P\cdot C\cdot d_{p}\cdot d_{c}+R\cdot P\cdot C\cdot d_{c}\right)$$

This design ensures computational efficiency by reducing transformer depth to 6 layers and replacing the feedfor- ward classifier with a capsule network, which enhances feature representation without significant complexity overhead.

### Model evaluation

To evaluate the performance of our text classification model, we employ the following metrics:

• *Recall* measures the overall correctness of the model:
  (23) Recall=TP/(TP+FN)
• *F1-Score* is the harmonic mean of precision and recall, and is particularly useful for imbalanced datasets:
  (24) $$F1_{score}=\left(2\cdot TP\right)/\left(2\cdot TP+FP+FN\right)$$

Since the dataset is imbalanced, we emphasize the use of F1-Score and Recall as more reliable metrics for evaluating model performance in such scenarios.

## Method validation

### Dataset

#### SWMH

The Self-Reported Mental Health (SWMH) [9] shown in Table 1 is a collection of Reddit posts used for identifying different mental health conditions. It contains 15,753 posts taken from five subreddits: r/depression, r/bipolar, r/anxiety, r/SuicideWatch, and r/offmychest. Each post typically includes multiple sentences where users talk about their feelings and personal experiences. The dataset is carefully curated to ensure quality and is used for multi-class classification of mental health issues.

**Table 1:** The data distribution across the train, validation, and test sets for each dataset used.

Dataset	Platform	Train	Validation	Test	Total
**SWMH**	Reddit	34,823	8706	10,883	54,412
**Dreaddit**	Reddit	2270	568	715	3553
**SAD**	SMS-like	5548	617	685	6850

#### Dreaddit

The Dreaddit dataset, introduced by Turcan and McKeown [10], in Table 1 focuses on detecting stress in Reddit posts. It includes 3553 posts from ten subreddits covering topics like financial difficulties, mental health issues, and personal conflicts. Each post consists of a short narrative where users describe their thoughts or situations. Posts are labeled as stressful or not stressful, based on human judgment. To ensure accuracy, each post is reviewed by five different annotators via Amazon Mechanical Turk, and the final label is assigned by majority vote. Posts with unclear or inconsistent labels are excluded.

#### SAD

The SAD dataset, created by Mauriello et al. [11], in Table 1 is designed to identify the cause of stress in short messages. It contains 6850 SMS-style sentences, each categorized into one of nine stress-related areas, such as school, finance, family issues, health, and emotional problems. These categories are adapted from the well-known Holmes and Rahe Stress Scale. The data was collected from a chatbot system and further expanded by adding similar messages for underrepresented categories. Labels were assigned through two rounds of human annotation using Amazon Mechanical Turk, making it a reliable dataset for studying the sources of everyday stress.

### Comparative study

Table 2 presents the performance comparison of several transformer-based models on the SWMH [9], Dreaddit [10] and SAD [11] datasets, evaluated using Recall and F1-Score. These metrics were selected for two primary reasons. First, to maintain consistency with the evaluation protocol of prior work whose models we extend [3]. Second, Recall is particularly important in mental health prediction tasks, as correctly identifying positive cases is critical. Meanwhile, F1-Score is well-suited to handle class imbalance, providing a more balanced view of model performance.

**Table 2:** Performance comparison on various datasets.

Model	SWMH[9]	Dreaddit [10]		SAD [11]		Time Complexity
	Recall F1-Score	Recall F1-Score		Recall F1-Score		
BERT	69.78	70.46	78.46	78.26	62.77 62.72	O 10(n^2^d + nd^2^)
RoBERTa	70.89	72.03	80.56	80.56	66.86 67.53	O 10(n^2^d + nd^2^)
BioBERT	67.10	68.60	75.52	74.76	66.72 66.71	O 10(n^2^d + nd^2^)
ClinicalBERT	67.05	68.16	76.36	76.25	62.34 61.25	O 10(n^2^d + nd^2^)
MentalBERT	69.87	71.11	80.28	80.04	67.45 67.34	O 10(n^2^d + nd^2^)
MentalRoBERTa	70.65	72.16	81.82	81.76	68.61 68.44	O 12(n^2^d + nd^2^)
**MentalRoBERTa(6) + Capsule Layer**	**71.72**	**71.68**	**80.97**	**80.86**	**65.47 65.22**	O 6(n^2^ d + nd^2^) + nddp + PCdp dc + RPCdc
**MentalRoberta(6) + Capsule Layer(Data augmentation)**	**71.91 72.08**	**81.49 81.28**	**65.92 65.71**	O 6(n^2^d +nd^2^) + ndd_p_ + PCd_p_d_c_ + RPCd_c_		

Our proposed model, **MentalRoBERTa(6) + Capsule Layer**, exhibits robust performance across multiple mental health classification tasks while maintaining computational efficiency. On the **SWMH** dataset, the model achieves a Recall of **71.72** and F1-Score of **71.68**; on **Dreaddit**, it reaches a Recall of **80.97** and F1-Score of **80.86**; and on **SAD**, it records a Recall of **65.47** and F1-Score of **65.22**. Incorporating **data augmentation** further enhances these results, boosting performance to a Recall of 71.91 and F1-Score of 72.08 on SWMH, Recall of 81.49 and F1-Score of 81.28 on Dreaddit, and Recall of 65.92 and F1-Score of 65.71 on SAD. The key advantage of our architecture lies in its improved computational efficiency. By employing a reduced stack of six MentalRoBERTa layers combined with a Capsule Layer, the model significantly lowers the computational cost compared to the full 12-layer MentalRoBERTa model, without sacrificing predictive accuracy. The resulting time complexity is: $(6\cdot \left(n^{2}\cdot d+n\cdot d^{2}\right)+n\cdot d\cdot d_{p}+P\cdot C\cdot d_{p}\cdot d_{c}+R\cdot P\cdot C\cdot d_{c})\;$making it more suitable for real-world deployment where inference speed and resource efficiency are critical.

However using **data augmentation** with **Bert** gives a small boost in classification performance than **Synonym Replacement**, but it also increases the time needed for data generation and training because the dataset becomes larger. So, we use original data in our experiments, as our focus is making the model faster and more efficient for real-world use.

### Layered architecture

**Fig. 3:**
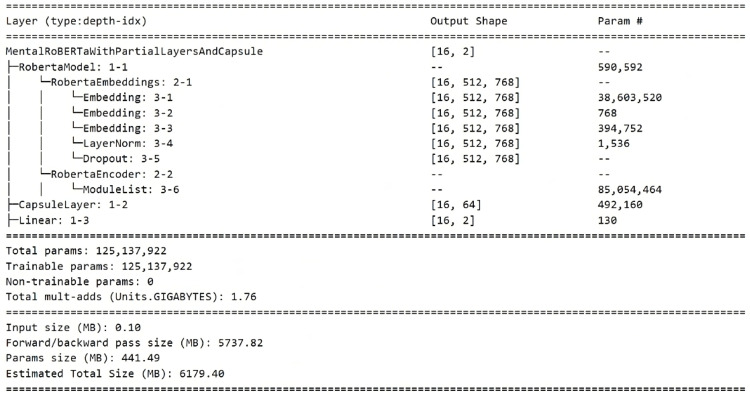
Visual summary of the proposed model for mental illness detection from social media text.

The architecture of the MentalRoBERTa With Partial Layer And Capsule model is built on top of a pre-trained MentalRoBERTa backbone, followed by a capsule layer and a final linear classifier shown in Fig. 3. The Roberta Model section includes an embedding layer and a stack of encoder layers, specifically utilizing a subset of layers as specified (e.g., 6 out of 12). The Capsule Layer reduces the high-dimensional output of the encoder’s [CLS] token to a more compact 64-dimensional representation using dynamic routing principles. This output is passed to a Linear layer for final classification into two output classes for second dataset with batch size 16. The model contains approximately 125.1 million trainable parameters, with an estimated memory footprint of **∼**1.15 GB, making it both powerful and relatively efficient for downstream mental health detection tasks.

### Error analysis using confusion matrix

**Fig. 4:**
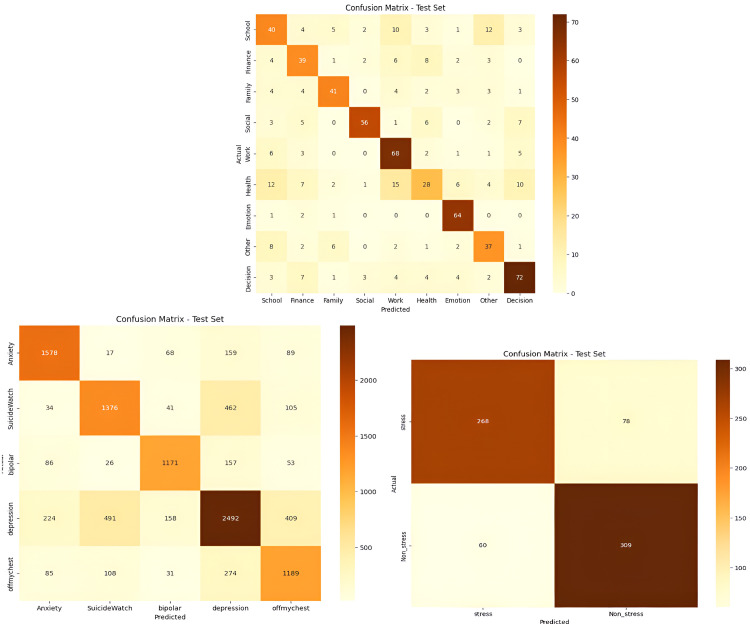
Visual comparison of confusion matrix across datasets.

The confusion matrices in Figs. 4 offer detailed insights into the classification performance of the proposed **MentalRoBERTa(6) + Capsule Layer** model.

In the multi-class mental health classification setting (Dataset 1), the model demonstrates strong predictive capability overall—particularly for the depression class, with 2492 true positives. However, a closer inspection reveals considerable misclassification between depression, SuicideWatch, and ofmychest, with 462 depression posts predicted as SuicideWatch and 274 as ofmychest. This misclassification is not surprising given the semantic and emotional overlap between these categories. For instance, users discussing suicidal ideation often express symptoms of depression, and similarly, posts tagged under *ofmychest* frequently contain emotionally vulnerable content that may not be clinically distinct from depression or anxiety. From a linguistic perspective, these posts share overlapping patterns of affectively charged language, expressions of hopelessness, or calls for emotional support, making it challenging even for humans to clearly delineate them without additional context. The model, despite being pretrained on domain-specific data, still operates within this ambiguity and is likely to confuse classes where boundaries are fuzzy and context is nuanced. Furthermore, these errors may be amplified by imbalanced class distributions and the lack of explicit relational encoding between classes in traditional softmax-based classifiers. While capsule networks do improve spatial and hierarchical representation of features, they are still susceptible to confusion when semantic classes are only subtly differentiated. In the topic classification task (Dataset 3), similar issues persist. Although the model performs well in high-signal classes like work and social, it shows noticeable overlap among school, family, and decision-making topics. This likely arises because many posts involve intertwined discussions—for instance, a decision about school often relates to family expectations or financial stress—resulting in ambiguous textual signals that the model may distribute across multiple classes. Without task-specific disambiguation mechanisms (e.g., topic disentanglement modules), the model struggles to isolate dominant themes in mixed-content posts. The binary stress detection task (Dataset 2) shows a more balanced outcome, with 268 true positives and 309 true negatives. Yet, the 78 false positives and 60 false negatives point to a sensitivity issue. This likely stems from the subjective nature of stress expressions, where subtle cues (e.g., sarcasm, passive aggression, or internalized anxiety) may not be strongly represented in the training data or may fall outside the model’s learned boundaries. Posts labeled as *non-stress* may still include stress-indicative language, but without explicit labeling, the model finds it difficult to draw precise boundaries.

Despite these challenges, the overall performance remains strong, demonstrating that the combination of domain-adapted RoBERTa and capsule layers provides a solid foundation for understanding emotional and thematic nuance. However, the observed misclassifications highlight the need for more robust post-processing strategies, such as:

Hierarchical classification, Contrastive learning, label refinement techniques such as expert annotation review, semi-supervised learning, ambiguity handling strategies like uncertainty-aware modeling and confidence thresholding to better manage overlapping linguistic cues (e.g., between depression and suicidal ideation).

### Model explainability with LIME

**Fig. 5:**
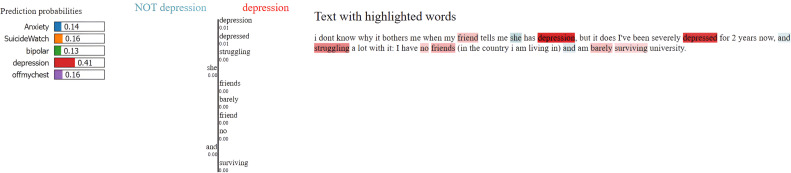
Visual of model preditions using LIME on depression class on dataset 1.

**Fig. 6:**
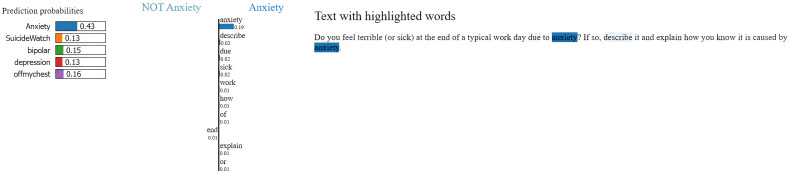
Visual of model preditions using LIME on anxiety class on dataset 1.

**Fig. 7:**
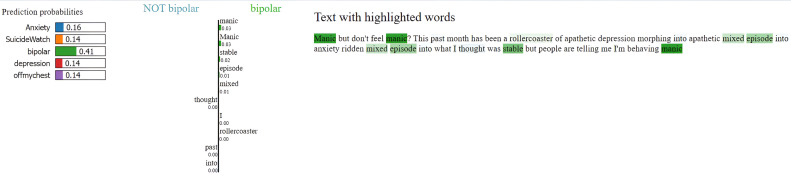
Visual of model preditions using LIME on bipolar class on dataset 1.

**Fig. 8:**
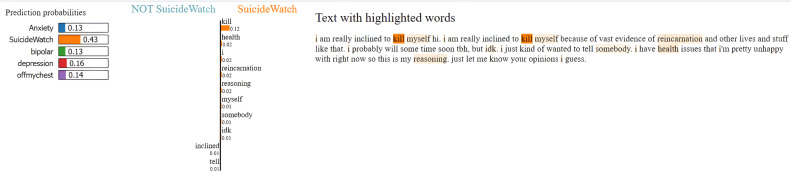
Visual of model preditions using LIME on suicidewatch class on dataset 1.

**Fig. 9:**
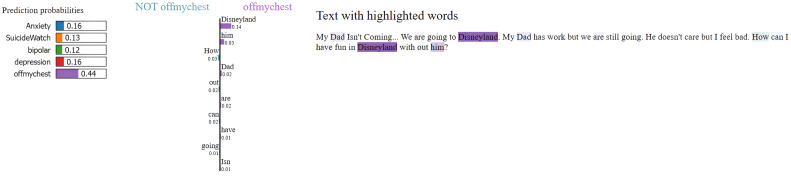
Visual of model preditions using LIME on offmychest class on dataset 1.

To enhance transparency and interpretability of the MentalRoBERTa(6) + Capsule Layer model, we incorporated LIME (Local Interpretable Model-agnostic Explanations) as a core component of our evaluation process. LIME was crucial for understanding and visualizing the model’s decision-making, particularly in a sensitive domain like mental health where opaque predictions can have serious consequences. It allowed us to generate word-level attributions that revealed whether the model’s predictions were based on psychologically meaningful cues or confounding, emotionally neutral terms. For example, in Fig. 5, Fig. 6, Fig. 7, Fig. 8, Fig. 9(Dataset1), LIME showed that correct classifications often relied on emotionally charged or clinically relevant words like “kill myself” or “episode,” while misclassifications occurred when attention shifted to irrelevant tokens like “Disneyland” or ambiguous words like “name.” This interpretability enabled in-depth error analysis, especially among closely related classes such as depression and SuicideWatch, and helped identify cases where the model over-relied on surface-level lexical patterns or failed to capture subtle linguistic nuances like sarcasm or implicit emotional cues. Additionally, LIME supported ethical and regulatory goals by increasing model transparency, aligning with frameworks such as GDPR that emphasize the right to explanation, and enhancing user trust—especially in clinical or support-tool deployment scenarios. Beyond explanation, insights from LIME guided model refinement, including suggestions for improving attention mechanisms and loss reweighting strategies. Overall, LIME served not just as a visualization aid but as a diagnostic, validation, and model improvement tool that significantly contributed to the robustness and reliability of our system.

## Limitations

While the proposed MentalRoBERTa(6) + Capsule Layer architecture demonstrates strong performance across both multi-class and binary mental health classification tasks, several limitations should be acknowledged. First, the datasets used—SWMH and Dreaddit—are derived from Reddit, which may not represent broader or more diverse populations. Linguistic expressions, mental health disclosures, and social norms on Reddit can differ significantly from those in clinical or less-anonymous settings, thus limiting the generalizability of the model. To partially address this, we also include the SAD dataset, which consists of short, SMS-like messages. This helps us evaluate the model’s performance on more personal, direct forms of communication, offering a different linguistic structure and context than Reddit posts. However, differences in platform-specific language and intent still present challenges in developing universally robust models. Second, despite employing performance metrics such as Recall and F1-Score to handle class imbalance, some categories (e.g., ofmychest) still experience relatively higher misclassification rates. Employing more sophisticated strategies could help mitigate this issue. Third, although LIME was utilized to provide insights into model behavior, it offers only local and approximate explanations. These interpretations may not fully capture the deeper, hierarchical semantics learned by the capsule network, and could occasionally mislead in cases involving sarcasm, irony, or nuanced psychological states. Addressing these limitations in future work could lead to a more robust, interpretable, and ethically responsible framework for mental health detection and support.

## Ethics statements

All data used in this study was previously collected by other researchers and sourced from existing datasets. The authors did not conduct any new data collection or direct interaction with individuals. All datasets were either publicly available or obtained from third-party researchers under conditions ensuring full anonymization. No personally identifiable information was accessed by the authors, and informed consent was handled by the original data collectors where required. Therefore, no additional consent procedures were necessary for this study.

## CRediT authorship contribution statement

**Faheem Ahmad Wagay:** Conceptualization, Methodology, Visualization, Investigation, Writing – original draft. **Jahiruddin:** Supervision, Writing – review & editing. **Yasir Altaf:** Visualization, Writing – review & editing, Validation.

## Declaration of competing interest

The authors declare that they have no known competing financial interests or personal relationships that could have appeared to influence the work reported in this paper.

## Data Availability

Data will be made available on request.
